# A Review of the Work of Dr. Jo E Frencken: The Pioneer of Atraumatic Restorative Treatment for Dental Caries

**DOI:** 10.7759/cureus.74546

**Published:** 2024-11-26

**Authors:** Saudamini More, Laresh Mistry, Amit Patil, Bhavani Baddi, Jyotsna Sethumadhavan, Anamika Sinha

**Affiliations:** 1 Public Health Dentistry, Bharati Vidyapeeth (Deemed to be University) Dental College and Hospital Navi Mumbai, Mumbai, IND; 2 Paediatric Dentistry, Bharati Vidyapeeth (Deemed to be University) Dental College and Hospital Navi Mumbai, Mumbai, IND; 3 Conservative Dentistry and Endodontics, Bharati Vidyapeeth (Deemed to be University) Dental College and Hospital Navi Mumbai, Mumbai, IND; 4 Oral Pathology, Bharati Vidyapeeth (Deemed to be University) Dental College and Hospital Navi Mumbai, Mumbai, IND; 5 Prosthodontics, Bharati Vidyapeeth (Deemed to be University) Dental College and Hospital Navi Mumbai, Mumbai, IND

**Keywords:** dental caries, "historical vignette", minimally invasive procedure, preventive dentistry, public health interventions

## Abstract

Dr. Jo E Frencken is a stalwart in preventive dentistry and cariology. He has worked extensively in diagnosing, treating, and assessing outcomes related to dental caries. He developed the concept of a novel technique known as atraumatic restorative treatment (ART) in 1986 for caries management. ART for field settings, where only hand instruments are used for caries removal, is a benchmark discovery made in the field of dentistry. His idea of ART has been adopted by the World Health Organization and has been included as a part of the Basic Package for Oral Care (BPOC) and practiced globally. He has dedicated his research to dental public health, caries prevention, and treatment modalities in various settings for over 40 years.

## Introduction and background

Dr. Jo E Frencken, a Dutch oral health scientist, is a pioneer of atraumatic restorative treatment (ART) in dentistry, which was developed in the year 1986. He has been unceasingly working for almost 40 years, constantly updating his knowledge in the literature [1]. 

Dr. Frencken is currently the head of the Department of Global Oral Health at Radboud University in Nijmegen, Netherlands. His areas of expertise include dental caries management, atraumatic restorative treatment, composites, fluorides, preventive dental procedures, and dental public health. Frencken has authored numerous articles and papers on ART and related topics, contributing significantly to the scientific literature in the field of cariology and restorative dentistry. He is an active researcher with over 250 publications to his credit. Frencken has been involved in training dental professionals worldwide on the implementation of ART, ensuring that the methodology can be effectively used in diverse settings [2].

The present review was planned to understand how Dr. Frencken's research paved the way for contemporary practice in preventive and restorative dentistry. This review highlights his work in minimal intervention dentistry and atraumatic restorative dentistry in particular.

## Review

Dr. Frencken, the pioneer of ART, came up with a simple yet minimally invasive technique for the management of caries, which did not require an airotor for caries removal. His idea is used extensively for caries management by dental clinicians belonging to various specialties, including orthodontic patients, vulnerable groups, and children [3].

Atraumatic restorative treatment

The advent of the ART approach was 37 years ago in Tanzania. It has evolved into an essential conservative caries management technique for facilitating accessible oral health care worldwide. ART gained international recognition and was adopted in various countries as a practical approach for caries management, especially in areas with limited access to advanced dental care [4].

Concept

The ART approach entails the use of hand instruments exclusively for removing carious tooth structure and then restoring the cavity using a conventional glass polyalkenoate (ionomer) restorative cement (GIC). Glass ionomer cement shows numerous biomimetic properties, which include the release of fluoride ions, pulpal protection, and chemical bonding to tooth substance [2]. Currently, ART is defined as ‘a minimally invasive care approach in preventing dental caries and stopping its further progression. It consists of two components: sealing caries-prone pits and fissures and restoring cavitated dentine lesions with sealant-restorations [3].

Rationale

This technique was conceptualized by Dr. Frencken with the idea of making treatment for dental caries in children more accessible. The primary motive for this treatment was to provide treatment at outdoor locations where electricity may not be available and to allow for the caries process to be arrested. The technique also came in handy for patients with dental anxiety primarily caused by the sound of airotor [4].

History

The ART approach was first pioneered in Tanzania where 28 teeth were treated in an outreach project. Carious tooth structure was removed by hand excavators and filled with glass ionomer cement. Only one tooth of those teeth that were treated needed extraction. The rest of the teeth were pain-free and functional for up to nine months. Dr. Frencken presented these findings at an international meeting in Dar es Salaam, Tanzania [4].

ART survival analysis research

A common argument that needed to be addressed was the quality and longevity of glass ionomer cement restoration provided in atraumatic restorative treatment. The studies done from 2006 to 2012 concluded that the 2-year-old survival rate for single-surface ART restoration (94.3%) was superior to multiple surface ART restorations (65.4%) in primary posterior teeth. In primary dentition, it was proven that the survival rates for single-surface and multiple-surface ART restorations during the initial 2 years were 93% (CI, 91-94%) and 62% (CI, 51-73%), respectively. Whereas in permanent dentition, for single-surface ART restorations during the initial 3 and 5 years, it was 85% (CI, 77-91%) and 80% (CI, 76-83%), respectively; that in multiple-surface ART restorations in permanent teeth at the end of 1 year was 86% (CI, 59-98%) [5]. The 3-year and 5-year survival rates in permanent posterior teeth were 87.1% and 77%, respectively. This was reported in a meta-analysis done in 2018 [5].

Acceptability, Feasibility, and Patient Satisfaction With ART

ART has been well-received among patients, especially those with special needs, because it is less invasive than traditional methods, requiring no anesthesia or electrical dental instruments. However, some dental professionals express hesitancy toward ART, often viewing it as a lower-quality alternative compared to conventional methods. Despite this perception, studies have shown that patients, including those with disabilities, tend to find ART more acceptable due to its less intimidating and more comfortable process [6].

ART is especially feasible in clinical settings where traditional restorative techniques may be challenging, such as in rural areas, or for patients with behavioral or intellectual difficulties. Research has demonstrated that ART restorations can be completed successfully in these environments, making it a cost-effective and accessible treatment option [7].

Patient satisfaction with ART has been generally high. The less invasive nature of the procedure, combined with its relatively quick and pain-free process, leads to positive patient experiences. For individuals with disabilities, ART often represents a more manageable treatment option, resulting in improved compliance and follow-up care [7].

Pain, Discomfort, and Anxiety Related to ART

When compared to conventional restorative treatment modalities, ART is a minimally invasive technique using only hand instruments, which avoids the need for drills and often eliminates the need for anesthesia. This contributes to lower pain and discomfort levels for both children and adults, making it a preferable option for patients with dental anxiety or fear [8].

Research highlights that ART generally causes less anxiety and discomfort than traditional restorative methods. This is due to the absence of noise and vibration from dental drills, which are common triggers of dental anxiety. ART has been particularly effective in children and anxious adults, helping alleviate the fear often associated with dental visits by minimizing painful stimuli​ [8].

However, some studies suggest that dental anxiety levels may not differ significantly across different treatment groups (traditional vs. ART), and factors like age, gender, and psychological state play important roles. Further research is encouraged to fully understand these variables [9].

Oral Health-Related Quality of Life Among Patients Treated With ART

Dr. Frencken’s work on atraumatic restorative treatment extends beyond clinical effectiveness and highlights its impact on oral health-related quality of life (OHRQoL) [10]. OHRQoL is a measure of how oral health affects a person’s overall well-being, including their comfort, appearance, and ability to eat, speak, and socialize without pain or embarrassment [11].

Dr. Frencken has contributed to an assessment of oral health-related quality in children treated with atraumatic restorative treatment. The trial was done among six to seven-year-old schoolchildren to compare three treatment protocols for managing cavitated carious dentine lesions: conventional treatment, atraumatic restorative treatment, and ultraconservative treatment. Oral health-related quality of life in children was assessed after three years of implementation of the trial. It was done for treating cavitated carious dentine lesions in primary molars and assessed using the Brazilian Early Childhood Oral Health Impact Scale. It was found that the perception of OHRQoL by parents/caregivers was similar in all the treatment protocols [10].

Overall, ART has shown great potential in improving patient care, particularly in underserved populations, and is seen as an effective alternative to conventional treatment in many cases. However, ongoing efforts are necessary to overcome professional skepticism and further validate its long-term benefits [10].

Contribution to Basic Package for Oral Care

The population in middle and low-income countries has limited access to dental care. A huge gap exists between oral health needs and care utilization in these countries. After the Alma Ata Declaration in 1976, this was the first conscious effort to provide ‘Health for All’ in terms of oral health care. With this in view, the basic package for oral care was launched [12]. World Health Organization’s Collaborating Centre (Nijmegen) developed ‘the Basic Package of Oral Care (BPOC)’. The package outlines sustainable basic oral care activities which can be readily provided by existing first line care of the Primary Health Care System. Dr Frencken was one of the founder members of this initiative. The BPOC consists of three basic elements: (a) Affordable fluoride toothpaste, (b) Atraumatic restorative treatment, and (c) Urgent oral care [13].

ART sealants

The primary purpose of a sealant is to create a cleansable occlusal surface by blocking plaque retentive deep pits and fissures. An ART sealant was introduced by Dr. Frencken with the idea of excavating caries using hand instruments followed by placing high-viscosity glass ionomer cements in caries-prone teeth. The primary indications for using ART sealants are similar to regular sealants, which include past caries experience in primary dentition, deep pits and fissures, active enamel carious lesions, and operator experience [14].

A meta-analysis of the studies done from 2005 to 2010 with the aim of assessing the survival rates of ART sealants. The survival rates of single-surface sealants were 93% (CI, 91-94%) and multiple-surface ART restorations 62% (CI, 51-73%) in primary teeth during the initial 2 years. The survival rate during the first 3 and 5 years for single-surface restorations in permanent teeth was 85% (CI, 77-91%) and 80% (CI, 76-83%), respectively. The survival rate over 1 year for multiple-surface ART restorations was 86% (CI, 59-98%) in permanent dentition [15].

Work on dental caries in children

Dr. Frencken has worked extensively in treating early carious lesions in children and minimal intervention techniques [16]. A study done in Peru showed that dental caries affected the quality of life at early ages, especially cavitated lesions and those with pulpal involvement [17]. A parameter studied by him with his co-workers is the level of anxiety with regard to atraumatic restorative treatment. The research found that dental anxiety scores were lower both in child and adult patients treated by ART than in those who received traditional restorative treatments [18]. He has reviewed various instruments for the detection of caries in epidemiological studies. It was recommended that calculations pertaining to carious lesions should be reposted as the presence of cavitated dentine carious lesions and the prevalence of enamel carious lesions be reported separately [19].

Dr. Frencken also introduced a novel concept of ART sealants. ART sealants are high-viscosity glass ionomer cements that are placed on teeth having caries-prone pits and fissures. The instruments used include an excavator and an applier/carver for adjusting the bite and removing excess material. They were primarily indicated in deep pits and fissures and teeth having carious enamel. This technique showed acceptable retention rates [14].

He has worked with Dr. Avijit Banerjee on caries excavation for minimally invasive techniques, which is highly referred to in clinical techniques and highly cited in caries research. A recommendation was made about selective and stepwise removal of carious tooth structures rather than complete or non-selective removal of dentine to preserve tooth structure [20]. A few recommendations were also made with regard to caries removal by hand instruments in cavitated lesions. For teeth with shallow or moderately deep lesions, selective removal to firm dentine is recommended, while in deep lesions, selective removal to soft dentine should be done [21].

Notable work for orthodontic cases

Dr. Frencken's expertise lies primarily in minimally invasive approaches for caries management, but his specific contributions to caries management in orthodontic cases deal with the application of atraumatic restorative treatment (ART) for restoring primary teeth in the arch for space management [22].

A novel treatment protocol was developed for treating small carious lesions known as the ultra-conservative treatment (UCT) by Gomide et al. This was a combination approach where small cavities were restored by the atraumatic restorative technique and a toothbrush and fluoride toothpaste were used to clean large open cavities. Casts treated with the UCT treatment protocol did not differ significantly from the conventional restorative technique. The earlier exfoliation of UCT-treated primary molars did not worsen the eruption pattern of permanent successors [22].

Dr. Frencken has co-authored research on primary molars done in Brazil among children. They evaluated whether restoring primary teeth using an atraumatic restorative technique negatively impacted space management (displacement). It was concluded that there was no negative impact on space management and ART could be effectively used for restoring teeth, especially in low and middle-income countries. The application of his principles in orthodontics helps ensure effective caries control while minimizing the impact on the ongoing orthodontic process [23].

Work in HIV-related oral health lesions

The human immune virus has documented effects on oral health. Dr. Frencken has worked in collaboration with researchers from Kenya. They have worked on training 800 primary health care worker providers to perform an oral examination. This intervention was made to integrate early recognition and management of oral lesions, the frequency of early detection of HIV-related oral lesions, and referral rates for HIV testing at health facilities [24]. They also tested the effect of a health promotion model in a similar setting [25].

Awards and recognition

Dr. Frencken is globally recognized as the father of atraumatic restorative treatment for dental caries management. He was awarded International Dentist of the Year in 1999. Jo E. Frencken is also awarded China's prestigious International Scientific and Technological Cooperation Award in 2016 [26]. He has authored the book titled ‘The Art and Science of Minimal Intervention Dentistry and Atraumatic Restorative Treatment’ [27].

Beyond his research and clinical work, Dr. Frencken has had a profound impact on dental education. His efforts to train dental professionals worldwide in the principles and practice of ART have shaped curricula in many countries. Internationally, ART is now a standard component of dental education, ensuring that future generations of dentists are equipped with the knowledge and skills necessary to offer minimally invasive, accessible treatments. His influence extends to shaping WHO guidelines and promoting global oral health strategies, cementing his legacy in the field of dental public health [28,29].

## Conclusions

Dr. Frencken has created a revolution in modern dentistry by establishing the atraumatic restorative technique, a combination of preventive and curative treatment procedures. It is an affordable and minimally invasive procedure. ART is now being used as a regular training program for dentists/oral health professionals in many countries. He has also contributed to the early detection of oral lesions in high-risk groups of HIV patients in underprivileged parts of the world.

Dr. Frencken’s ART methodology is part of a broader trend in minimally invasive dentistry, which emphasizes the preservation of healthy tooth structure and aims to reduce overtreatment. Alongside techniques such as selective caries removal and the use of preventive sealants, ART is increasingly recognized as a cornerstone of patient-centered, conservative dental care. This movement aligns with global health initiatives that prioritize preventative over curative approaches to healthcare.
